# Age Differences in Executive Control Assessed with a Robotic Object Hit and Avoid Test of Rapid Motor Decision Making

**DOI:** 10.1093/geroni/igaa057.3255

**Published:** 2020-12-16

**Authors:** Alexandra Watral, Kevin Trewartha

**Affiliations:** Michigan Technological University, Houghton, Michigan, United States

## Abstract

Motor decision-making processes are required for many standard neuropsychological tasks, including the Trail Making Test (TMT), that aim to assess cognitive functioning in older adults. However, in their standard formats, it is difficult to isolate the relative contributions of sensorimotor and cognitive processes to performance on these neuropsychological tasks. Recently developed clinical tasks use a robotic manipulandum to assess both motor and cognitive aspects of rapid motor decision making in an object hit (OH) and object hit and avoid (OHA) task. We administered the OH and OHA tasks to 77 healthy younger adults and 59 healthy older adults to assess age differences in the motor and cognitive measures of performance. We administered the TMT parts A and B to assess the extent to which OHA performance is associated with executive functioning in particular. The results indicate that after controlling for hand speed, older adults performed worse on the OH and OHA tasks than younger adults, performance declines were far greater in the OHA task, and the global performance measures, which have been associated with cognitive status, were more sensitive to age differences than motor measures of performance. Those global measures of performance were also associated with measures of executive functioning on the TMT task. These findings provide evidence that rapid motor decision making tasks are sensitive to declines in executive control in aging. They also provide a way to isolate cognitive declines from declines in sensorimotor processes that are likely a contributing factor to age differences in neuropsychological test performance.

